# Universal versus targeted coronavirus disease 2019 (COVID-19) arrival antigen testing on subsequent COVID-19 infections in military trainees

**DOI:** 10.1017/ash.2024.365

**Published:** 2024-10-22

**Authors:** Marquise D. Westbrook, James Aden, John W. Kieffer, Erin L. Winkler, Angela B. Osuna, Theresa M. Casey, Dianne N. Frankel, John L. Kiley, Heather C. Yun, Joseph E. Marcus

**Affiliations:** 1 Infectious Diseases Service, Department of Medicine, Brook Army Medical Center, JBSA-Fort Sam Houston, TX, USA; 2 Biostatistics Department, San Antonio Uniformed Services Health Education Consortium, JBSA-Fort Sam Houston, TX, USA; 3 Trainee Health Surveillance, 559^th^ Medical Group, JBSA-Lackland, TX, USA; 4 USAFRICOM HQ, Office of the Command Surgeon, Stuttgart, GE, USA; 5 Department of Medicine, Uniformed Services University, Bethesda, MD, USA

## Abstract

In this retrospective cohort study of military trainees, symptomatic-only coronavirus disease 2019 (COVID-19) arrival antigen testing decreased isolation requirements without increasing secondary cases compared to universal antigen testing. Symptomatic-only arrival antigen testing is a feasible alternative for individuals entering a congregant setting with a high risk of COVID-19 transmission.

## Introduction

Congregate settings are at particularly high risk for coronavirus disease 2019 (COVID-19) transmission, and the best practices for arrival screening in these settings to prevent transmission are not known. Guidelines for COVID-19 screening do not recommend for or against antigen testing for congregate settings due to unclear benefits and risks of false-negative tests leading to the inadvertent introduction of the virus to a susceptible population.^1^ Previous work in the United States Air Force (USAF) Basic Military Training (BMT) demonstrated decreased isolation requirements with universal COVID-19 arrival antigen testing compared to universal nucleic acid amplification testing without a subsequent increase in secondary cases or case clusters.^2^ As symptomatic USAF BMT trainees were most associated with future case clusters in military training groups, entry COVID-19 testing was changed from universal antigen testing to symptomatic-only antigen testing in October 2021.^2,3^ This study evaluates the impact of BMT COVID-19 entry testing with symptomatic-only antigen testing on isolation requirements, development of secondary cases, and case clusters.

## Methods

All individuals who reported for BMT during the period of August 1, 2021–December 15, 2021, when the delta variant was predominant in the United States, were included in this retrospective cohort study.^4^ Trainees lived in groups of 35–50 individuals in open bay dorms with minimal exposure to individuals outside the training base for 7.5 weeks of training. Between August 1, 2021, and October 27, 2021, all trainees were given a standardized symptom screening form and underwent universal antigen screening via nasopharyngeal swab using BinaxNOW™ COVID-19 Ag Card by Abbott (Scarborough, ME). Between October 27, 2021, and December 15, 2021, trainees were given a standardized symptom screen and only underwent antigen testing if they had any symptoms consistent with COVID-19. Trainees with a positive antigen test were isolated for 10 days before returning to their respective training group for the entirety of the study period. A secondary case was defined as a trainee who did not test positive for COVID-19 upon arrival but tested positive between days 2 and 14. A case cluster was defined as 5 or more cases in 1 training group, as previously described.^2,3^ During all time points in the study period, non-pharmaceutical interventions remained in effect as previously described.^2^

Universal antigen testing was compared to symptomatic-only testing based on the number of trainees who tested positive on arrival, number who developed secondary cases, and day of the secondary case. Furthermore, the number of training groups with cases on arrival, with a secondary case, and with a case cluster was also compared. Nominal variables were compared by χ^2^ or Fisher’s exact test, as appropriate. Continuous variables were compared by the Mann-Whitney test. A *P*-value < .05 was considered statistically significant.

The 59th Medical Wing Institutional Review Board (IRB) deemed this protocol (IRB number FWH20200092N) as public health surveillance; therefore, informed consent was waived.

## Results

During the study period, 13,447 trainees presented to BMT, and 305 (2.3%) tested positive for COVID-19 during the first 2 weeks of training. Universal testing resulted in significantly more trainees testing positive (.6% vs .1%, *P* = <.0001) on arrival, compared to symptomatic-only testing (Table 1). The number of symptomatic arrivals was greater in the universal screening compared to the symptomatic-only screen (.3% vs .1%, *P* = .01). Approximately half of the trainees (53%) who tested positive in the universal antigen testing group were symptomatic. There was no difference in secondary cases between the testing strategies (1.9% vs 1.7%, *P* = .4). There was no significant difference in the median days from arrival to a secondary case in the universal testing group compared to the symptomatic-only testing (median, 12; IQR, 9–13 vs 13 [13–14]; *P*-value < .00001).

**Table 1.** Impact of coronavirus disease 2019 arrival screening on trainees who arrived between August 1 and December 15, 2021

	Universal testing (n=8505)	Symptomatic-only testing (n=4942)	*P*-value
Positive during Day 1–14	217 (2.5%)	88 (1.8%)	0.005
Positive on arrival	53 (0.6%)	5 (0.1%)	<0.0001
Asymptomatic positive on arrival	25 (47%)	0	0.06
Positive on Day 2–14	161 (1.9%)	83 (1.7%)	0.4
Median days until secondary case	12 [9–13]	13 [13–14]	<0.0001

Of the 193 training groups that underwent universal screening 21 (11%) had a positive test on arrival compared to 5 of 107 (5%) that underwent symptomatic-only testing (Table 2). There was no difference in number of training groups with secondary cases (21% vs 19%, *P* = .7). There was also no difference in the number of training groups that developed case clusters (5% vs 7%), *P* = .6) between the testing strategies.

**Table 2.** Impact of coronavirus disease 2019 (COVID-19) arrival screening on COVID-19 cases by training group, August 1–December 15, 2021

	Universal testing (n=193)	Symptomatic-only testing (n=107)	*P*-value
Training groups with positive case on Arrival	21 (11%)	5 (5%)	0.09
Training groups with case on Day 2–14	40 (21%)	20 (19%)	0.7
Training groups with >5 cases	10 (5%)	7 (7%)	0.6

## Discussion

COVID-19 testing is crucial for basic military trainees as they live in large, open bay dorms, where close proximity and shared facilities increase the risk of virus transmission leading to subsequent isolation. In this observational study of 13,447 military trainees, symptomatic antigen testing resulted in fewer trainees being isolated for COVID-19 without differences in secondary cases or number of training groups having a secondary case of COVID-19.

COVID-19 involves a wide degree of presentations that range from asymptomatic to critical illness. Previous work in the BMT population has shown that with each variant, the presenting symptoms of COVID-19 have changed.^5^ As this intervention relies on symptom-based screening, it is essential for future efforts to have a wide case definition so that the diverse presentations of COVID-19 are accounted for. There were more symptomatic trainees isolated in the cohort with universal screening compared to the symptom-only screening (.3% vs .1%), which may be due to a reporting bias in trainees with more mild symptoms when symptomatic-only testing was occurring. As national case rates were similar between the 2 time points, it is unlikely that the explanation is due to less circulating COVID-19 in the community.^6^

There are several limitations to this study. First, there are different viral characteristics that affect testing with each COVID-19 variant, and it is unclear how well this data from the delta wave applies to other variants.^7^ Second, symptoms were self-reported and may have been inconsistently reported or not reported if self-attributed to another cause to avoid potential isolation for a highly motivated population. Vaccination rates were not available for this study. Finally, this study evaluated a young military population, who were prescreened for medical comorbidities and conclusions should not be extended to other congregate settings.

In this observational study of 13,447 trainees, universal arrival antigen testing resulted in significantly more trainees requiring isolation at the beginning of training without a change in the number of COVID-19-positive cases or case clusters in the first 2 weeks of training. Symptomatic antigen testing for individuals entering congregate settings is a feasible alternative to universal testing for individuals about to enter a congregant setting with a high risk of COVID-19 transmission.
